# Supplementary material for: Association between Mutuality and Health-Related Quality of Life in Patient–Caregiver Dyads Living with Schizophrenia
Source: Int J Environ Res Public Health. 2021 Mar 2;18(5):2438. doi: 10.3390/ijerph18052438 (PMC7967568; doi:10.3390/ijerph18052438)
Supplement: Supplementary file 1 [file ijerph-18-02438-s001.pdf]

Supplementary Materials

Table S1. Characteristics of dyads of patients with schizophrenia and caregivers (*n*=133).

Characteristic	Mean	SD	n	%[b]
**Patient's characteristics**				
Age (years)	45.39	9.35		
Gender				
Male			75	56.4
Female			58	43.6
Marital status				
Unmarried[a]			113	85
Married			20	15
Occupation				
Unemployed or retired			101	75.9
Employed			32	24.1
Education				
Junior high school or below			49	36.8
Senior high school/diploma			66	49.6
Bachelor or above			18	13.5
Length of mental illness (years)	16.42	7.43		
Number of psychiatric hospitalizations	5.99	3.14		
Severity of psychiatric symptom	8.48	4.03		
**Caregiver's characteristics**				
Age (years)	54.98	11.84		
Gender			46	34.6
Male			87	65.4
Female				
Relationship to the patient				
Parent			59	44.4
Spouse			9	6.8
Sibling			43	32.3
Child/close relative or friend			22	16.5
Marital status				
Unmarried[a]			47	35.3
Married			86	64.7
Occupation				
Unemployed or retired			53	39.8
Employed			80	60.2
Education				
Junior high school or below			86	64.7
Senior high school/diploma			30	22.6
Bachelor or above			17	12.8
Monthly income (NTD)				
25,000			69	51.9
≥ 25,000			64	48.1

Table S2. Mutuality and health-related quality of life (HRQoL) in the dyads of patients and caregivers (n = 133): Differences and relationships.

	**Scale scores**		**Differences**		**Relationships**	
	Patients	Caregivers	Between Dyads		Between Dyads	
Scale	Mean (SD)	Mean (SD)	*T*	*p*	*r*	*p*
Mutuality	2.12 (1.04)	1.69 (0.89)	5.6	< .001	0.59	< .001
HRQoL						
Total score	86.34 (16.08)	89.75 (15.32)	-2.51	.013	0.56	< .001
Domains						
Physical health	12.79 (2.49)	13.65 (2.74)	-3.12	.002	0.26	.003
Psychological health	12.23 (2.43)	12.33 (2.28)	-0.42	.672	0.26	.002
Social relationships	11.82 (2.93)	12.96 (2.28)	-4.57	< .001	0.41	< .001
Environment	12.15 (2.9)	12.62 (2.24)	-2.16	.033	0.54	< .001

Note: SD = standard deviation; *r* = Pearson's product-moment correlation coefficient.
